# Influence of Light on Plant–Phyllosphere Interaction

**DOI:** 10.3389/fpls.2018.01482

**Published:** 2018-10-12

**Authors:** Sofia D. Carvalho, José A. Castillo

**Affiliations:** ^1^Colegio de Ciencias Biológicas y Ambientales, Universidad San Francisco de Quito, Quito, Ecuador; ^2^School of Biological Sciences and Engineering, Yachay Tech University, Urcuquí, Ecuador

**Keywords:** phyllosphere, biotic interactions, plant–microbe interactions, light, narrow-bandwidth wavelengths, LEDs

## Abstract

Plant–phyllosphere interactions depend on microbial diversity, the plant host and environmental factors. Light is perceived by plants and by microorganisms and is used as a cue for their interaction. Photoreceptors respond to narrow-bandwidth wavelengths and activate specific internal responses. Light-induced plant responses include changes in hormonal levels, production of secondary metabolites, and release of volatile compounds, which ultimately influence plant–phyllosphere interactions. On the other hand, microorganisms contribute making some essential elements (N, P, and Fe) biologically available for plants and producing growth regulators that promote plant growth and fitness. Therefore, light directly or indirectly influences plant–microbe interactions. The usage of light-emitting diodes in plant growth facilities is helping increasing knowledge in the field. This progress will help define light recipes to optimize outputs on plant–phyllosphere communications. This review describes research advancements on light-regulated plant–phyllosphere interactions. The effects of full light spectra and narrow bandwidth-wavelengths from UV to far-red light are discussed.

## Introduction

The phyllosphere, this complex and relatively unknown world of microbes interacting among themselves and with plant hosts, particularly with the aerial organs, is the subject of this review. The phyllosphere is not a closed system but is affected by abiotic factors that influence and shape its dynamics. Light is one important abiotic factor. Light directly impacts plants and microorganisms as both groups harbor photoreceptors. Light also exerts indirect effects such as modification of moisture on leaf surfaces, change of microbial lifestyle, and habits, alteration of phyllosphere composition and diversity among others. The current review summarizes findings on this triple interaction: plant–microbe–light; and describes advances on the promotion of plant growth elicited by microorganisms driven by energy collected from light in its different wavelengths.

For many years, research on symbiotic microorganisms and their relationship with plants has mostly focused on microorganisms residing in the roots of plants, that is, the rhizosphere. Much less was known about phyllosphere comparing to rhizosphere. However, many investigations on phyllosphere have been reported lately due to the massive production of data resulting from the use of omics and related techniques. This has driven a significant advance in the understanding of microbial dynamics in the aerial organs of plants, mainly in leaves. The participation of light in this interaction is a matter of recent studies too. Specific light wavelengths impact these interactions to different extents. The increasing usage of light emitting-diodes, which emit narrow bandwidth wavelengths, in research laboratories and plant growth facilities is setting the basis to unravel light-regulated plant–phyllosphere interactions. The purpose of this review is to provide an independent look at the current work of the light effect on plant–phyllosphere interaction. Effects of narrow bandwidth wavelengths from UV to far-red light are described with an emphasis on details arising from specific plant hosts and microorganisms. Knowledge to be completed in future research is discussed.

## Phyllosphere, Definition and Components

The term phyllosphere refers to the community of microorganisms that live under symbiotic relationship with plants, particularly on the leaves, stems, buds, flowers, that is, in the aerial parts of the plant. These microorganisms live both on surfaces of plant organs (usually referred as phylloplane) or inside plant tissues (endosphere). This community is composed by bacteria, viruses, fungi, algae, archaea, and infrequently by protozoa and nematodes (Vorholt, 2012). Bacteria surpass by far other groups, both in number of cells and diversity of taxonomic groups (Andrews and Harris, 2000). The phyllosphere constitutes the largest concentration of microorganisms on earth, after the soil habitat, since the leaf surface of terrestrial plants is estimated to exceed 6.4 × 10^8^ square kilometer worldwide. Considering that the density of bacterial cells on leaf surfaces reaches 10^6^–10^7^ cells per square centimeter, the total amount of bacteria may exceed 10^26^ cells, without counting other taxonomic groups sharing same habitat (Lindow and Brandl, 2003). Most of the bacterial groups are scarcely known or are undescribed species as revealed by recent metagenomics studies (Lambais, 2006; Knief et al., 2012). Although the aerial surfaces are often an inhospitable environment for microorganisms, because it is an open system highly influenced by fluctuating abiotic conditions and poor nutrient availability, microbial species have managed to colonize this environment. Successful interactions play pivotal role on the homeostasis of plants and offer some benefits like promotion of plant growth, defense against pathogens and in general driving plant performance to cope with different stresses (Venkatachalam et al., 2016; Saleem et al., 2017).

Early studies of the phyllosphere microorganisms focused on plant pathogens (Montarry et al., 2008). However, since the vast majority of microorganisms are commensal on their host plants, more widespread and deeper studies were initiated (Rastogi et al., 2013). New technologies based on massive sequencing have allowed performing culture-independent analyses that raised great opportunities for characterizing phyllosphere diversity, ecological properties, physiology/metabolism, and beneficial outcomes. These analyzes are opening new areas of study that integrate not only plant–microbe relationship, but also environmental factors such as humidity, temperature, radiation, etc. Phyllosphere microbiota is thus an important field for studying the diversity, interrelation, and flow of energy and resources within microbial communities and between microorganisms and the host plant (Lindow and Brandl, 2003).

Phyllosphere interactions begin with the dispersal of the inoculum from different sources, including air, rainwater, soil, insect vectors, seeds, and even animal feces. The initial inoculum arrives at the plant organ and proceeds to establish itself on the surface. This step usually depends on the plant genotype and may be organ specific (Knief et al., 2010). In this case, the plant exerts a strong selection for community composition in the early stages of colonization and then proceeds to generate different community assemblies influenced by the spatial associations between plants (Maignien et al., 2014). For most plants, air or wind is an important source of inoculum. Microorganisms can also arrive on aerial organs via rainfall and subsequent splashing of raindrops which can be effective carriers of microorganisms into plants (Vacher et al., 2016; Joung et al., 2017). Insects also play a significant role on transferring the inoculum, since there is evidence that they harbor a large number of microbes on their body surfaces, as well as in their intestines, which are transferred to flower surfaces when the insects visit them (Ushio et al., 2015) or feed sap (Ghanim et al., 2017). Transmission through seeds, also known as “vertical inoculum,” represents another alternative for colonization and establishment, although its contribution is only partial requiring additional sources of inoculum because usually the diversity found on the host plant is higher than that in the seeds (Lopez-Velasco et al., 2013).

Following the establishment, adhesion or motility proceeds. Since the majority of phyllosphere microorganisms are commensal, they usually do not activate the immune defenses of the plant. Considering that the surface conditions of leaves and other aerial organs are harsh, phyllosphere microorganisms tend to form biofilms to protect themselves from aggressive environmental factors (Morris et al., 1997). Some compounds produced by leaves help microbial colonizers to support their metabolism. Bacteria acquire amino acids, which are useful as nitrogen source and few other molecules rich in carbon. Although carbon seems to be less available than nitrogen on the leaf surface, some bacteria (*Methylobacterium*) have managed to use plant-released methanol as carbon and energy source (Knief et al., 2012). Microbial groups unable to use methanol have developed other strategies like the production of indole-3-acetic acid (IAA), which loosens plant cell walls and stimulates the release of simple sugars (Fry, 1989; Lindow and Brandl, 2003). Alternatively, some bacteria obtain energy from light. They produce specialized proteins (bacteriorhodopsin) that allow light capture and its conversion in chemical energy (Atamna-Ismaeel et al., 2012a).

## Promotion of Plant Growth by the Phyllosphere

The interaction of plants with microorganisms can be classified in three major categories: positive (symbiotic and productive interactions), negative (in which one or both parties are harmed by the other as in the pathogenesis), and neutral irrelevant interactions. As described above, plants provide microorganisms with a suitable – somehow difficult – habitat and sources of nitrogen and carbon for their metabolism. On the other hand, microorganisms provide protection to plants from pathogens by different mechanisms: niche competition, antimicrobials production, release of secondary metabolites, and induction of systemic resistance that stimulates plant defenses to face an eventual pathogen attack (Li et al., 2012; Lopez-Velasco et al., 2012; Kefi et al., 2015; Saleem et al., 2017). Likewise, microorganisms are involved in the promotion of plant growth through the production of growth regulators (Venkatachalam et al., 2016) and making available certain essential nutrients like nitrogen, phosphorus, and others (see below; Fu et al., 2016).

There are many reports showing that microorganisms in the rhizosphere and phyllosphere produce natural growth regulators, such as auxins, that enhance plant growth and therefore the increase of nutrient uptake via root elongation (in the case of rhizosphere microorganisms), and photosynthesis activity by expanding the effective leaf area (in the case of phyllosphere microorganisms) (Mwajita et al., 2013). Although plant regulators produced by phyllosphere microorganisms are indirectly harnessed by plants, they constitute an additional or complementary input to the normal plant hormonal pool. The most common plant regulator is the auxin IAA, which elicits and regulates growth and development in plants (Teale et al., 2006). Different groups of microorganisms from the phyllosphere, such as bacteria and fungi, possess IAA-producing capabilities similar to those of plants, either using tryptophan or not as a precursor (Spaepen et al., 2007; Sun et al., 2014; Venkatachalam et al., 2016; Thapa et al., 2018). An important amount of phyllosphere microorganisms show the capability to produce IAA. As a result, the microbe-produced IAA is able to effectively induce plant growth and promote overall health, which makes it an attractive alternative to synthetic auxins in agricultural production (Adesemoye and Kloepper, 2009; Hayat et al., 2010).

Phyllosphere microorganisms also intervene in the metabolism of some nutrients for the plant. Some groups of leaf-associated microorganisms are able to fix nitrogen (N) from atmospheric sources. In this process, they produce organic N using the highly specialized enzyme called nitrogenase. Plants then absorb the organic N as a complement to satisfy their internal demands. The dominant bacterial groups of the phyllosphere are diazotrophic (nitrogen-fixing) bacteria according a number of studies (Kembel et al., 2014; Lambais et al., 2017). Phyllosphere microorganisms are also involved in modifying nutrients to make them available for plant uptake. A well-known process is the solubilization of phosphorus (P). As being found in natural reservoirs as inorganic and rock-like form, P is converted to plant assimilable form through a process that involves organic acid production and chelation (Chen et al., 2006; Mohammadi, 2012). The phosphate-solubilizing microorganisms enhance plant growth mainly in environments with deficiency in P by solubilizing insoluble phosphates commonly found in the soil. However, phosphate-solubilizing microorganisms are not only found in the rhizosphere but also in the phyllosphere. A number of recent studies have shown that an important percentage of bacterial isolates sampled from phyllosphere were able to solubilize phosphates (Mwajita et al., 2013; Batool et al., 2016). P is commonly found in soils and, to a lesser extent, on plant surfaces as inorganic element, therefore its bioavailability is reduced. The strategy employed by P-solubilizing microorganism is the production of low molecular weight organic acids that chelate cations found in phosphate making it soluble for plant uptake (Goldstein, 1995; Rodrìguez and Fraga, 1999). The acidic conditions favor the fixation of P by free oxides and hydroxides of aluminum and iron, increasing the efficiency of P incorporation on plants cells and tissues. This process is complex and usually involves the physiological condition of bacteria that keep close interaction with plants and their released compounds (Reyes et al., 1999).

Another element that is essential for almost all living organisms is iron. This element is physiologically important since a great number of proteins require iron for their activities, particularly the enzymes involved in redox reactions (Scavino and Pedraza, 2013). Although iron is very common on the earth crust (Israel Science and Technology, 2018), it is poorly available for biological utilization. One strategy employed by organisms to obtain available iron is the production and usage of siderophores. Siderophores are low molecular weight organic molecules that show great affinity for iron-based nutrients and increase their mobility and availability (Ahmed and Holmström, 2014). Siderophores are usually released to extracellular environments and once bound to iron, the resulting siderophore–iron complex is collected and taken into the cell by specific siderophore channels or receptors (Saha et al., 2013). Both, plants and microbes are able to produce siderophores under iron limiting conditions (Morrissey and Guerinot, 2009; Scavino and Pedraza, 2013). However, in plants most of the iron is acquired from rhizosphere microorganisms. For this, plants differentially express genes involved in metal transport and chelation (in the epidermis) and in sensing and control iron levels (in the vasculature) (Morrissey and Guerinot, 2009). Although, the most important source of iron for plants are rhizosphere microorganisms, it has been proven that phyllosphere microorganisms also contribute with siderophores and acquisition of this element. Thapa et al. (2018) have demonstrated that IAA and siderophore producers were the dominant microbial groups living on rice leaves. Similarly, Fu et al. (2016) showed that 15 strains isolated from the phyllosphere and rhizosphere were able to produce siderophores, being *Pseudozyma aphidis* JYC356 the strain with the highest siderophore-producing capacity. From a biotechnological point of view, the use of siderophore-producing microorganisms appears as an alternative to control some pathogens. For example, the soybean phyllosphere-living *Pseudomonas syringae* pv. *syringae* strain 22d/93 showed the capability of controlling *P. syringae* pv. *glycinea*, through an indirect way that employs siderophores, which enhances the fitness and competitiveness of the controlling strain (Wensing et al., 2010).

## Factors Affecting Phyllosphere–Plant Interactions

Both environmental and plant-dependent factors contribute to shape microbial communities in the phyllosphere. Regarding abiotic stresses, these microorganisms must cope with day/night regimes (that create a wide range of temperature fluctuations), exposure to sunlight (which includes UV radiation), availability of water and moisture, presence of wind and salt. On the other hand, the multilayer conformation of leaf tissues shapes different physical spaces that are occupied by microorganisms. This structure affects interaction of microorganisms with plants and between them. In particular, the access to moisture, nutrients, gas (O_2_, CO_2_, etc.) and other molecules must be adjusted by microbial cells in the different microhabitats molded in the leaf concavities. Physiological stages of plants as well metabolic status, especially the availability of some carbohydrate compounds, directly affect surviving (Trouvelot et al., 2014). As mentioned above, some nitrogen-based nutrients provided by plants are important for microorganisms, however, its availability is not regular. Moreover, microbial communities show a dynamic performance according to seasons or the plant life cycle. Shade et al. (2013) demonstrated the constant community changes before flower opening through flower senescence. Lopez-Velasco et al. (2013) showed data on the increasing complexity of phyllosphere microorganisms throughout the life of spinach leaves. In this case, they found a richer community in seedlings compared to seeds or cotyledons. However, microbial structure is not always consistent, because it can vary at temporal, developmental, and spatial scales (Delmotte et al., 2009).

## The Role of Light

In contrast to the rhizosphere, phyllosphere microorganisms do not only take advantage by exchanging chemical compounds with the host plant but also benefit from light. Some bacterial groups contain light harvesting bacteriorhodopsins which were previously reported exclusively in aquatic systems (Atamna-Ismaeel et al., 2012b). Atamna-Ismaeel et al. (2012a) found that microbial rhodopsin captures a different fraction of light that does not interfere with the light spectrum absorbed by their host plant. These groups are aerobic anoxygenic phototrophs that might use light as a complementary source of energy (Vorholt, 2012; Stiefel et al., 2013).

Plants possess photoreceptors that sense narrow-bandwidth light wavelengths and activate specific internal responses. Mediated by the activity of these sensors, light is a major regulator of plant growth and development much beyond photosynthesis. Light controls a broad range of aspects at the level of gene expression, metabolism, and whole plant physiology, including responses to other abiotic factors and to biotic stimuli (Folta and Carvalho, 2015).

**FIGURE 1:**
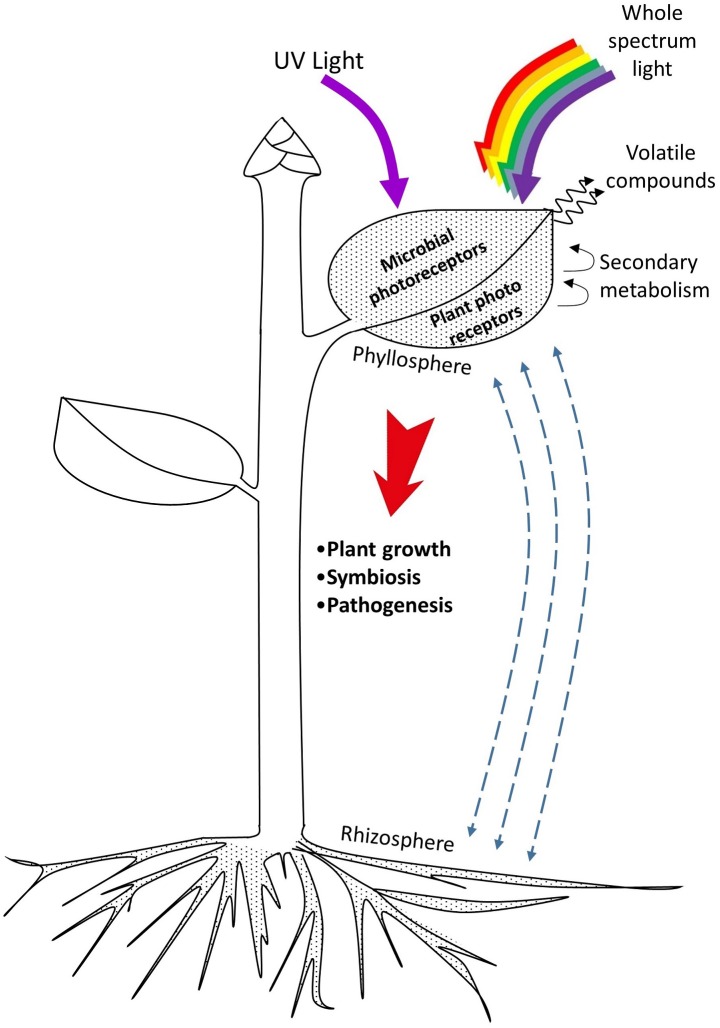
Plant–phyllosphere interactions influenced by light. Microbial residents of leaves and other aerial plant organs interact with rhizosphere microorganisms by exchanging individuals and other compounds. Both plant and microbial photoreceptors capture UV and other components of the light spectrum to perform a variety of metabolic functions. Close interactions between plants and microorganisms are mediated by molecules from primary and secondary metabolism produced by plants, and by important organic-type elements and plant growth regulators released by microorganisms. These interactions ultimately define plant growth and fitness.

Specific light environments also influence the establishment of the phyllosphere (**Figure 1**). This effect has been explored in research for decades. Nowadays plant–phyllosphere interactions may be targeted with selective colored cover surfaces on greenhouses that modify the indoor light spectra, as well as with artificial lighting. The latter appears as a solution for higher control. Light emitting-diodes (LEDs), in particular, emit narrow-bandwidth wavelengths and allow the construction of specific light environments to precisely target behaviors of the phyllosphere, plant development, and both aspects combined. A knowledge with the potential of being introduced in plant growth facilities for a cleaner control of deleterious biotic factors and a promotion of beneficial plant–phyllosphere interactions (Carvalho and Folta, 2014; Alsanius et al., 2017). However, this is a complex field under development given that plant–phyllosphere–light interactions are plant species- and microorganism-specific, and dependent on day-night length and light quality and intensity. The majority of studies have focused on the role of UV light, but evidence shows that the whole solar spectrum is important (**Tables 1–4**).

**Table 1:** Direct light effects on phyllospheric microorganisms, with examples of individual microorganisms and effects of light sources.

	Direct light effects on microorganisms	
	
	Microorganism	
	
Light source	Species (Reference)	Effect
White	*Pseudomonas* sp. DR 5-09 (Gharaie et al., 2017)	Changing capacity for substrate utilization
	*Botrytis cinerea* (Canessa et al., 2013)	Conidia formation
	*Neurospora crassa* (Canessa et al., 2013)	Conidia formation
UV	Multiple reports (Rastogi et al., 2010)	DNA damage, production of ROS
Blue	*Pseudomonas* sp. DR 5-09 (Gharaie et al., 2017)	Changing capacity for substrate utilization
	*Botrytis cinerea* (Canessa et al., 2013)	Conidia formation
	*Cercospora zeae* (Kim et al., 2011a)	Biosynthesis of cercosporin
	*Fusarium graminearum* (Kim et al., 2014)	Conidia formation
	*Neurospora crassa* (Ballario et al., 1996)	Conidia formation
	*Peronospora effusa* (Choudhury and McRoberts, 2017)	Sporangial germination
	*Trichoderma atroviride* (Cetz-Chel et al., 2016)	Conidia formation
Red	*Pseudomonas* sp. DR 5-09 (Gharaie et al., 2017)	Changing capacity for substrate utilization
	*Peronospora belbahrii* (Cohen et al., 2013)	Inhibition of sporulation


*For additional examples see text.*

**Table 2:** Direct light effects on plant–phyllosphere interactions, with examples of plant host–microorganism interactions and effects of light sources.

	Direct light effects on microorganisms	
	
	Host–microorganism interaction	
	
Light source	Example (Reference)	Effect
White	Rose-*Podosphaera pannosa* (Suthaparan et al., 2010a)	Reduction of number of spores
UV	Peanut-*Bacillus coagulans* (Jacobs and Sundin, 2001)	Predominance under UV exposure
	Peanut-*Clavibacter michiganensis* (Jacobs and Sundin, 2001)	Predominance under UV exposure
	Peanut-*Curtobacterium flaccumfaciens* (Jacobs and Sundin, 2001)	Predominance under UV exposure
	Rice-*Enterobacter cloacae* (Kumar et al., 2016)	Predominance under UV exposure
	Cucumber-*Podosphaera xanthii* (Suthaparan et al., 2014)	Suppression of powdery mildew
	Rose-*Podosphaera pannosa* (Suthaparan et al., 2012)	Suppression of powdery mildew
Blue	Maize-*Cercospora zeae* (Kim et al., 2011a)	Synchronization pathogenesis-maize photoperiodic responses
Red	Basil-*Peronospora belbahrii* (Cohen et al., 2013)	Inhibition of sporulation
	Broad bean-*Botrytis cinerea* (Islam et al., 1998)	Inhibition of hypha formation and infection
	Onion-*Botrytis cinerea* (Islam et al., 1998)	Inhibition of hypha formation and infection
	Rose-*Podosphaera pannosa* (Suthaparan et al., 2010b)	Suppression of powdery mildew
R:FR	Rose-*Podosphaera pannosa* (Suthaparan et al., 2010b)	Reduced suppression of powdery mildew by far-red


**Table 3:** Plant-mediated light effects on plant–phyllosphere interactions, with examples of light-induced plant traits that affect microorganism behavior.

	Plant-mediated light effects on microorganisms
	
Light source	Plant responses (Reference)
White	Emission of volatile compounds (Gouinguene and Turlings, 2002)
UV	Primary and secondary metabolites (Mewis et al., 2012; Li et al., 2017; Heinze et al., 2018; Vandenbussche et al., 2018)
	Hormone pathways, JA and SA (Vandenbussche et al., 2018)
	Cell wall thickness (Demkura and Ballaré, 2012; Vandenbussche et al., 2018)
	Epicuticular wax (Young et al., 2012)
	GABA pathway (Balint-Kurti et al., 2010)
Blue	Secondary metabolites (Kim et al., 2013)
	Defense-related genes (Ahn et al., 2015)
	Antioxidant/antimicrobial capacities (Kim et al., 2013; Kook et al., 2013)
	Cell wall thickness (Kim et al., 2013)
	ROS metabolism (Cetz-Chel et al., 2016)
Red	Primary and secondary metabolites (Shirasawa et al., 2012; Parada et al., 2014)
	Hormone pathways, SA and cytokinins (Islam et al., 2008; Wang et al., 2010)
	Defense-related genes (Ahn et al., 2015)
	Antioxidant/antimicrobial capacities (Islam et al., 2002b; Khanam et al., 2005)
	ROS metabolism (Xu et al., 2017)
R:FR	Secondary metabolites (Cargnel et al., 2014)
	Hormone pathways, JA and SA (Cerrudo et al., 2012; de Wit et al., 2013; Leone et al., 2014; Gommers et al., 2017)
	Cell wall thickness (Shibuya et al., 2011)
Green	Defense-related genes (Nagendran and Lee, 2015)


*For additional examples see text. JA, jasmonic acid; SA, salicylic acid.*

**Table 4:** Plant-mediated light effects on plant–phyllosphere interactions, with examples of plant host–microorganism interactions affected by plant traits and effects of light sources.

	Plant-mediated light effects on microorganisms	
	
	Host–microorganism interaction	
Light source	Example (Reference)	Effect
White	Wheat-*Fusarium graminearum* (Ansari et al., 2014)	Control of defense gene expression to the mycotoxin deoxynivalenol
UV	Arabidopsis-*Botrytis cinerea* (Demkura and Ballaré, 2012)	Resistance to *Botrytis cinerea* via the sinapate pathway
	Maize-*Cochliobolus heterostrophu*s (Balint-Kurti et al., 2010)	High bacterial diversity and reduced resistance to Southern leaf blight disease
	Soybean-*Phakopsora pachyrhizi* (Young et al., 2012)	Resistance to *Phakopsora pachyrhizi* through epicuticular wax
Blue	Grapevine-*Botrytis cinerea* (Ahn et al., 2015)	Reduced development of gray mold disease
	Lettuce-*Botrytis cinerea* (Kook et al., 2013)	Reduced development of gray mold disease
	Tobacco-*Cucumber mosaic virus* (Chen et al., 2015)	Inhibition of virus spreading
	Tomato-*Botrytis cinerea* (Xu et al., 2017)	Reduced development of gray mold disease
Red	Arabidopsis-*Pseudomonas syringae* (Islam et al., 2008)	Resistance to *Pseudomonas syringae*
	Broad bean-*Botrytis cinerea* (Khanam et al., 2005)	Reduced development of gray mold disease
	Cucumber-*Sphaerotheca fuliginea* (Wang et al., 2010)	Resistance to powdery mildew
	Grapevine-*Botrytis cinerea* (Ahn et al., 2015)	Reduced development of gray mold disease
	Tomato-*Botrytis cinerea* (Xu et al., 2017)	Reduced development of gray mold disease
	Tobacco-*Cucumber mosaic virus* (Chen et al., 2015)	Inhibition of virus spreading
R:FR	Arabidopsis-*Pseudomonas syringae* (de Wit et al., 2013)	Decreased resistance to *Pseudomonas syringae* under low R:FR
	Arabidopsis-*Botrytis cinerea* (Cerrudo et al., 2012)	Increased susceptibility to *Botrytis cinerea* under low R:FR
	Basil-*Botrytis cinerea* (Elad et al., 2014)	Reduced gray mold incidence with increased plant spacing
	Cucumber-*Podosphaera xanthii* (Shibuya et al., 2011)	Reduction of powdery mildew under high R:FR
	Strawberry-*Botrytis cinerea* (Legard et al., 2000)	Reduced gray mold incidence with increased plant spacing
Green	Tomato-*Pseudomonas cichorii* (Nagendran and Lee, 2015)	Reduced disease caused by *Pseudomonas cichorii*


*JA, jasmonic acid; SA, salicylic acid.*

### White Light/Full Spectrum

The effect of the solar spectrum and artificial white light sources on plant–phyllosphere interactions has been addressed in open field and greenhouse-grown crops. Selective lighting in greenhouse has efficiently targeted fungal and bacterial microbiomes in sunflower plants (Alsanius et al., 2017). Abundance of fungal microbiome was highest under high-pressure sodium lamps, at intermediate levels under red/blue LEDs, and lowest in plants exposed to white LEDs. Species richness was overall not affected by specific light treatments, although slight changes in proportions were observed. Examples described *Ascomycota* representing 98.1% of the fungal population under white LEDs, and a reduced number to 93.5% under red/blue LEDs. The bacterial microbiome was overall less responsive to light treatments. The mechanisms governing these observations are not clear yet but some suggestions were reported. Fungi may be directly influenced by the physical properties of a particular light source, whereas bacteria seem to be affected indirectly through modification of the plant environment caused by the different light sources (Alsanius et al., 2017).

A direct effect of light on fungi is consistent with reported effects of white light on fungal conidiation and plant invasion by many fungi. Molecular studies have allowed the identification and characterization of different light-responsive elements with roles in light-mediated plant invasion, in species such as *Aspergillus, Botrytis, Neurospora, Sordaria, Candida*, and *Fusarium*. These light-responsive factors have specific functions in stimulating conidia formation (asexual spores) and virulence or the appearance of sclerotia (dormant structures) in light or darkness, via control of reactive oxygen species (ROS) homeostasis and secondary metabolism (Schumacher et al., 2014; Cohrs et al., 2016; Brandhoff et al., 2017; Wang et al., 2018). Increasing day lengths from 18 to 20–24 h with white florescent lamps may suppress severity of powdery mildew (*Podosphaera pannosa*) in rose due to a reduction in the quantity of spores produced and in the germination potential of conidia (Suthaparan et al., 2010a). A higher nutrient supply for fungal growth as a result of the prolonged photosynthetic activity of plants does not correlate, therefore, with a higher incidence of powdery mildew in rose. Light also affects circadian clock of fungi which in turn impact their fitness or virulence on plant host. A well reported case refers to the non-pathogenic fungus *Neurospora crassa* in which light plays a key role contributing to set up the circadian clock. In this case, the light cycle intervenes on modulating the outcome of the plant–pathogen interaction (Dunlap et al., 2007). For pathogenic fungi like *Botrytis cinerea*, light also regulates the circadian clock. Experiments designed to suppress the circadian rhythm by applying constant light or out-of-phase light:dark cycles result on the modification of Arabidopsis–*Botrytis* interaction (Hevia et al., 2015).

Establishing precise light treatments for a desired outcome may be possible with increased knowledge on plant–phyllosphere interactions at the molecular, metabolic, and hormonal levels. The Arabidopsis NPR1 (NONEXPRESSOR OF PATHOGENESIS-RELATED GENES 1) is a receptor for salicylic acid and a key regulator of systemic acquired resistance that confers broad-spectrum resistance (Wu et al., 2012). Transgenic expression of its homolog in rice targeted salicylic acid accumulation and resistance to *Xanthomonas oryzae* in a light intensity-dependent manner (Chern et al., 2005). NPR1 also mediates responses to the trichothecene mycotoxin deoxynivalenol (DON) produced by *Fusarium* in wheat. Expression of genes involved in defense to DON is white light-responsive and wheat genotype-dependent, indicating that light directly or indirectly influences plant defenses to DON (Ansari et al., 2014).

White light has also been used to target nutrient source on non-phototrophic phyllosphere elements. White LEDs on *Pseudomonas* sp. DR 5-09 affected respiratory profiles by changing capacity for specific substrate utilization (Gharaie et al., 2017). Carbon, nitrogen, and phosphorus sources were shifted in bacteria exposed to white light and other light conditions when compared to darkness. This observation proves that altered light environments may easily affect microbial lifestyle and habits of substrate usage by non-phototrophic phyllospheric microbiota, which in turn may interfere with phyllosphere composition and diversity (Gharaie et al., 2017).

Altered light environments in urban settings should also be considered as a factor that influences the phyllosphere. The artificial urban structure influences the radiation environment. The natural vegetation is replaced by concrete structures that interfere with light reflection and absorption by plants (Sieghardt et al., 2005). Anthropogenic pressures on trees can as well affect phyllosphere communities and their interaction with plant hosts (Mhuireach et al., 2016; Laforest-Lapointe et al., 2017). These effects are seen along gradients of population and infrastructure intensities. The composition of tree leaf bacterial and fungal communities in urban environments is, similarly to natural environments, influenced by the plant host, yet affected by the localization (Jumpponen and Jones, 2010; Smets et al., 2016; Laforest-Lapointe et al., 2017). Urban bacterial communities of the phyllosphere are dominated by *Alphaproteobacteria* but its relative abundance and composition decreases in more dense areas (Smets et al., 2016; Laforest-Lapointe et al., 2017). The role of the light spectrum has not yet been specifically addressed, but it may be possible that different densities of vegetation and built-up areas may affect ratios of incident light wavelengths on host plants with a direct effect on the phyllosphere. This fact may be considered in the development of strategies for the management of the urban forest, pest biological control in urban areas and in designing neighborhoods and open space systems that stimulate human health (Mhuireach et al., 2016; Laforest-Lapointe et al., 2017).

### UV Light

UV light has been the focus of most studies on effects of light on plant–phyllosphere interactions. UV radiation affects production of plant secondary metabolites, diversity of phyllosphere populations, orientation of pests toward plant hosts, and the behavior of biological control agents. Studies have focused on components of the phyllosphere such as fungi and bacteria. Specific ranges of UV light from UV-A to UV-C are of interest, and environments have been modified either by artificial light or selective covers that filter out fractions of the UV region. Short UV wavelengths may be toxic to microorganisms and other leaf dwellers, and thus the increasing efficiency of UV LEDs allows introducing the technology in crop systems for disease prevention or direct leaf surface sterilization (Wilson et al., 1999; Matsuura and Ishikura, 2014).

UV light triggers changes in plant tissues, such as the accumulation of secondary metabolites, that correlate with defense strategies and interactions with phyllosphere components (Demkura and Ballaré, 2012; Mewis et al., 2012). UV effects are dependent on light intensity and plant developmental stages (Moreira-Rodríguez et al., 2017). UV-A and UV-B can target different classes of primary and secondary metabolites, such as chlorophylls, carotenoids, phenolics, and glucosinolates, showing the potent effect of light on redirecting carbon flux in plants, with concomitant effects on the phyllosphere (Mewis et al., 2012; Li et al., 2017; Heinze et al., 2018; Vandenbussche et al., 2018). Accumulation of secondary metabolites is accompanied with increased expression of genes overlapping with the jasmonic acid and salicylic acid pathways, as seen by transcriptomics analyses of UV-B exposed Arabidopsis plants, suggesting activation of defense signaling mechanisms that may impact microbial communities (Vandenbussche et al., 2018).

Expression of cell wall modifying genes in Arabidopsis is responsive to UV-B light (Vandenbussche et al., 2018). The UV photoreceptor UVR8 in Arabidopsis senses UV-B and activates resistance responses to *B. cinerea* (Demkura and Ballaré, 2012). UVR8 controls the sinapate biosynthetic pathway but does not involve the activity of jasmonic acid pathway or the synthesis of tryptophan-derived antifungal compounds. Sinapates may be used as precursors for lignin synthesis and deposition, which has role in cell wall fortification against fungal invasion (Demkura and Ballaré, 2012). Another report has shown that altered levels of epicuticular wax upon differential UV light exposure also correlate with disease development of soybean rust caused by *Phakopsora pachyrhizi* (Young et al., 2012). Suppression of powdery mildew in cucumber and roses (*Podosphaera xanthii* and *Podosphaera pannosa*) upon UV-B exposure was, however, reported to result as direct damage by light on the pathogen and did not operate through the host (Suthaparan et al., 2012, 2014).

UV radiation directly influences bacterial structure and diversity in the phyllosphere. Peanut plants exposed to UV-B showed altered bacterial communities compared to control plants (Jacobs and Sundin, 2001). Organisms isolated under UV irradiation at the end of a growing season in Texas, United States, tended to show higher tolerance to UV, and predominant groups in peanut were *Bacillus coagulans, Clavibacter michiganensis*, and *Curtobacterium flaccumfaciens* (Jacobs and Sundin, 2001). Large-scale analysis in a UV-B resistant bacteria group isolated from rice, *Enterobacter cloacae*, revealed broad changes in ROS and in the number and expression of proteins upon exposure to UV-B (Kumar et al., 2016). Future studies in the function of these proteins, together with gene expression profiling, may further elucidate the mechanisms of phyllospheric bacteria resistance and adaptation to UV-B (Matallana-Surget and Wattiez, 2013; Kumar et al., 2016). Effects of UV light on bacterial diversity may as well impact other groups of microorganisms. In maize the diversity of leaf bacteria correlates with UV responses and with susceptibility to Southern leaf blight caused by the fungal pathogen *Cochliobolus heterostrophus* (Kadivar and Stapleton, 2003; Balint-Kurti et al., 2010). Analysis of quantitative trait loci (QTL) from maize with effects on bacterial leaf diversity and responses to UV-B identified a glutamate decarboxylase, part of the gamma-aminobutyric acid (GABA) biosynthetic pathway. High GABA levels as a result of UV-B exposure associate with high bacterial diversity in the phyllosphere. High bacterial diversity is in turn linked to lower maize resistance to Southern leaf blight disease. Leaf structural or metabolic properties may be at the basis of this correlation between low bacterial diversity and fungal resistance. Two explanations have been suggested: (1) maize leaves may restrict simultaneous colonization by both bacteria and fungi, with no interaction between bacteria and the pathogen; or (2) maize leaves encourage the development of suppressive bacterial species, leading to the inhibition of fungal growth under reduced bacterial diversity (Balint-Kurti et al., 2010).

Additional reports have described the impact of UV light on herbivores and insects, and their natural enemies. Plant secondary metabolites and the jasmonic acid pathway induced by UV light can serve as source of information for larvae and insects for host selection (Caputo et al., 2006; Demkura et al., 2010; Qi et al., 2018; Vandenbussche et al., 2018). Plant responses alter leaf traits to block attractiveness to herbivores and entrance on host cells (Caputo et al., 2006; Foggo et al., 2007; Mazza et al., 2013). On the other hand, reduction of UV light in plant growth facilities may disrupt visual cues that can decrease populations of insects in different crops (Díaz et al., 2006; Legarrea et al., 2012; Gulidov and Poehling, 2013; Elfadly et al., 2016). Competitors of insects were reportedly less affected than their targets by altered UV exposure, and UV-exposed plant tissues may be capable of releasing increased levels of volatile compounds that are more efficient in attracting parasitoids (Foggo et al., 2007; Legarrea et al., 2014; Dáder et al., 2015). Depending on specific target plant–phyllosphere interactions, strategies for plant growth may include moderate exposure of plants to UV-B at early growth stages to induce defense mechanisms and not affect plant growth and yield, and later suppression of UV light to restrict insect entrance and herbivore attack (Elfadly et al., 2016; Dáder et al., 2017). Each situation should be evaluated on a case-by-case basis, considering as well plant specific tolerance to UV irradiance and the tight control of UV conditions, including the spectrum provided by selected shading or light bulbs, and the intensity. In addition, combinations of UV light with variation in other abiotic factors, such as temperature, water availability, or salt levels, target plant fitness and increase complexities of plant–phyllosphere interactions (Ma et al., 2016; Escobar-Bravo et al., 2017).

### Blue Light

The White Collar Complex (WCC) is a transcriptional mediator of blue light effects on fungal invasion of plant leaves. WCC was first identified in *Neurospora crassa*, and later described in other fungi such as *B. cinerea, Aspergillus nidulans, Fusarium graminearum*, and *Magnaporthe oryzae* (Ballario et al., 1996; Kim et al., 2011b, 2014, 2015; Canessa et al., 2013; Liversage et al., 2018). WCC is formed by two GATA-type zinc transcription factors with LOV domains, WC-1 and WC-2 (White Collar-1 and White Collar-2). Blue light can repress conidia formation via WCC and other transcription factors and induce hyphae formation in *Botrytis*, and mediate responses to excessive light by targeting intracellular ROS levels (Islam et al., 1998; Canessa et al., 2013; Cohrs et al., 2016).

The increasing knowledge in this field may help create solutions for devastating fungi that cause major losses in important crops worldwide, such as *Magnaporthe oryzae* that affects rice, barley, wheat, pearl millet, and turf-grass, or *B. cinerea* that causes gray mold, and *Fusarium graminearum* that attacks wheat, barley, and corn and produces mycotoxins harmful to humans and animals (Kim et al., 2011b, 2014; Cohrs et al., 2016). This information has been assessed in crops such as tomato, lettuce, and grapevine, as blue light-irradiated leaves showed reduced development of gray mold disease (Kim et al., 2013; Kook et al., 2013; Ahn et al., 2015; Xu et al., 2017). Blue light may simultaneously inhibit *Botrytis* growth and affect plant properties that reduce fungi ability for leaf infection. These properties include the accumulation of proline and phenolic compounds, the expression of defense-related genes, higher antioxidant and antimicrobial capacities, and compact morphology, including cell wall thickness (Kim et al., 2013; Kook et al., 2013; Ahn et al., 2015).

Transcriptional networks and signaling cascades have revealed overlapping and divergent WCC mechanisms in different fungi, suggesting the existence of specific light responses that may have appeared with evolution (Canessa et al., 2013; Kim et al., 2014, 2015; Liversage et al., 2018). Many fungi exclusively infect leaves through natural openings, such as stomata. Crp1 (*Cercospora* regulator of pathogenesis) is a blue light photoreceptor in *Cercospora zeae* homologous to WC-1 required for the maize fungal pathogen to sense stomata (Kim et al., 2011a). Blue light triggers Crp1-mediated biosynthesis of cercosporin, a potent disruptor of host-cell membranes, allowing leaf infection through stomata openings. These observations together with the rhythmic opening and closure of leaf stomata suggest that *Cercospora zeae* uses light as a key environmental input to synchronize elements of pathogenesis with maize photoperiodic responses (Kim et al., 2011a). Specificity of blue light responses in fungi can also be seen with the stimulation by low blue light intensity of conidiation in *Trichoderma atroviride*. This effect is mediated by the Blu7 (Blue light up-regulated 7) transcription factor under control of the WCC counterpart, BLRC (Blue Light Regulator Complex). Blu7 acts on the cAMP pathway, ROS production, and nitrogen metabolism (Cetz-Chel et al., 2016). The complexity of fungal-plant-blue light responses underlies the importance of further exploring specific phyllosphere–plant interactions under detailed light environments, and identifying additional photoreceptors and signaling pathways that have not yet been unraveled.

Effects of blue light have been tested in other components of the phyllosphere, including oomycetes, bacteria, and virus. *In vitro* assays have shown reduction of sporangial germination of the oomycete *Peronospora effusa* upon exposure to blue light, when compared to red, green, and yellow light and dark, opening doors for a solution against spinach downy mildew, the most important threat to spinach production worldwide (Choudhury and McRoberts, 2017). In the non-phototrophic *Pseudomonas* sp. DR 5-09, blue light interfered with respiration capacity and substrate usage (Gharaie et al., 2017). This impairment was more evident under blue than white light, with the restriction of respiration of 140 substrates when compared to darkness. The high sensitivity of the tested strain to the blue portion of the spectrum suggests a strong regulatory activity of blue light receptors on internal pathways controlling nutrient usage. Blue light can also be used as an inhibitor of the interaction between *Nicotiana tabacum* and *Cucumber mosaic virus* (CMV). The replication of CMV has been reported to be delayed, levels of salicylic acid and cytokinin increased, and the activities of ROS-scavenging enzymes and antioxidative metabolism altered under blue light compared to white light (Chen et al., 2015).

### Red Light

Being one of the main promoters of photosynthesis and plant growth, red light, similarly to blue wavelengths, has been a target of high interest in studies of light effects on plant–phyllosphere interactions. Arabidopsis may be a model to be considered. In this plant, red light-induced resistance to the bacterial pathogen *Pseudomonas syringae* is mediated by systemic acquired resistance through a salicylic acid-dependent pathway (Islam et al., 2008). The same pathway seems to be at the basis of red light-induced resistance to powdery mildew in cucumber (Wang et al., 2010). Salicylic acid is, however, a suppressor of disease resistance to *B. cinerea* in broad bean plants (Khanam et al., 2005). Plant defense responses activated by internal signaling molecules, including salicylic acid, seem to depend on plant host and plant host-microorganism specificities. Thus, evidence suggests that red light can be used to target beneficial organisms and plant pathogens. However, the possibility of designing specific light environments for best outputs in these interactions needs further data at the molecular, hormonal, and physiological levels. For example, jasmonic acid is another important player in plant defense that interacts antagonistically with salicylic acid in dicotyledons but may share similar mechanisms in monocotyledons (Tamaoki et al., 2013; Caarls et al., 2015). Activity of red light on the jasmonic acid pathway, and on its crosstalk with salicylic acid, should therefore also be assessed.

Red light can be a solution to decrease incidence of fungal pathogens in different crops. It has been used in rice against brown spot disease caused by *Bipolaris oryzae* and blast disease caused by *Magnaporthe oryzae*. Red light induces synthesis of cinnamic acid, and resistance mediated by tryptophan and phenylpropanoid pathways, and may convert a host-pathogen compatible interaction into an incompatible one (Shirasawa et al., 2012; Parada et al., 2014). This red light effect seems to solely rely on the host plant and not on the behavior of the fungus. Host-specific light responses also seem at the basis of red-light induced delayed leaf spot by *Corynespora cassiicola* in cucumber, and red rich light sources can also decrease powdery mildew, caused by *Sphaerotheca fuliginea*, in this same crop (Schuerger and Brown, 1997; Rahman et al., 2010; Wang et al., 2010). Red light can also reduce gray mold incidence (*B. cinerea*) in grapevine, broad bean and tomato plants (Khanam et al., 2005; Ahn et al., 2015; Xu et al., 2017). Detached grapevine leaves exposed to red light showed differential expression of defense-related genes and increased accumulation of antimicrobial secondary metabolites such as stilbenic compounds, opening doors for a solution against pathogen infections together with alternative methods to enhance the accumulation of resveratrol in viticulture (Ahn et al., 2015). Broad bean resistance relied on increased antioxidant catalase activity, which may have role in scavenging endogenous H_2_O_2_ generated upon infection, and production of anti-fungal glycoproteins by plant cells (Islam et al., 2002b; Khanam et al., 2005). Altered ROS and antioxidant metabolism were reported in tomato, and red light seems to trigger different defense mechanisms than the ones described for purple light (Xu et al., 2017). Beyond communication with the host, red light inhibited hypha formation and infection by *B. cinerea* in onion and broad bean (Islam et al., 1998; Canessa et al., 2013).

Sporulation of *Peronospora belbahrii*, the cause of basil downy mildew disease, can be inhibited by exposing plants to red light (Cohen et al., 2013; Patel et al., 2016). Because *Peronospora belbahrii* sporulation occurs under darkness (López-López et al., 2014), exposure of basil plants to red light during nighttime may be an effective approach to reduce leaf damage and downy mildew disease incidence (Cohen et al., 2013). A short red light night-break may also be useful to suppress powdery mildew caused by *Podosphaera pannosa* in rose plants (Suthaparan et al., 2010b). Red light also increased resistance to *Phytophthora* blight, caused by *Phytophthora capsici*, in bell pepper, pumpkin and tomato seedlings (Islam et al., 2002a). Red light may, however, not be a solution for spinach downy mildew, as it increased the *in vitro* germination capacity of the oomycete (Choudhury and McRoberts, 2017).

Whereas white and blue light have the most profound effects in the respiratory profiles of *Pseudomonas* sp. DR 5-09, red light was the least effective light condition to impair substrate utilization, and was often similar to dark conditions (Gharaie et al., 2017). D-Galactose was the only carbon source with impaired utilization when compared to darkness. Red light was reportedly effective in reducing disease severity by *Pseudomonas cichorii* in tomato seedlings by upregulating defense-related genes, when compared to white light or darkness (Nagendran and Lee, 2015). Red light also negatively regulates CMV spreading in *Nicotiana tabacum* by targeting similar pathways than that of blue light (Chen et al., 2015).

### Far-Red Light

The effect of far-red light on plant–phyllosphere interactions has mostly been addressed in combinations with red light. Ratios of red to far-red light (R:FR) that signal neighbor proximity induce different physiological responses in plants, including to biotic factors. Low R:FR is sensed by phytochrome B and triggers a competition signal in plant canopies that suppresses plant immunity in shade-intolerant species (Moreno et al., 2009). The connection between R:FR and plant immunity may explain why most of studies have focused on pathogenic organisms. Future approaches must also look toward the phyllosphere beneficial diversity.

Studies in Arabidopsis have allowed establishing connections at the molecular level. Decreased resistance of Arabidopsis to *Pseudomonas syringae* under low R:FR is linked to reduced transcription of salicylic acid-responsive genes (de Wit et al., 2013). Increased susceptibility to *B. cinerea* under low R:FR results, in turn, from a decreased jasmonate sensitivity and is independent of salicylic acid (Cerrudo et al., 2012; de Wit et al., 2013). Altered jasmonate signaling results from the promotion of DELLA proteins degradation and the stabilization of the repressor of jasmonate sensitivity, JAZ10 (Leone et al., 2014). This mechanism reconfigures resource allocation and targets the reduction of the biosynthesis of tryptophan-derived secondary metabolites, including individual species of glucosinolates and the alkaloid camalexin (Cargnel et al., 2014).

Specific combinations of R:FR have been used to target other fungi and bacteria. Powdery mildew caused by *Podosphaera xanthii* has been reduced in cucumber seedlings acclimatized to high R:FR (Schuerger and Brown, 1997; Shibuya et al., 2011). Altered conidial development and fungal invasion seem to result from structural and non-structural modifications of leaves in response to high R:FR, such as thickened epidermal tissue, higher leaf mass per area, and altered secondary metabolism (Shibuya et al., 2011; Itagaki et al., 2016). Far-red light also reduced the red light-inhibition of powdery mildew in roses (Suthaparan et al., 2010b).

Knowledge from Arabidopsis has now been transferred to wild species. Two *Geranium* species with distinct native R:FR environments showed altered growth, transcriptomics and defense responses to *B. cinerea* when compared to far-red enriched locations (Gommers et al., 2017). Jasmonic acid-mediated responses were repressed by low R:FR in *Geranium pyrenaicum*, a species that occurs in open habitats and high R:FR, and increased in *Geranium robertianum*, which grows in a range of conditions such as forest understories where R:FR is low. The two species seem to have evolved different mechanisms related to plant immunity as a result of exposure to specific light environments and shade densities. R:FR-mediated immunity is controlled in *Geranium* by regulators that have not been identified in Arabidopsis (Gommers et al., 2017). This report highlights the importance of adaptation to particular habitats in ecological strategies and the establishment of plant–phyllosphere interactions.

Typical high-density plantings in agriculture may increase vulnerability of host plants to pathogens (Moreno et al., 2009). This low R:FR-mediated reduction of plant immunity may be overcome in crop fields through a tight control of planting density. Doubling spacing of sweet basil plants in the field reduced gray mold incidence and increased host resistance to pathogen infection with no significant yield losses (Elad et al., 2014). A similar approach targeting necrotrophic fungus has been described in strawberry but with reported yield losses (Legard et al., 2000).

### Green Light

The lack of identification of a green light receptor in plants has delayed studies on the effect of this region of the spectrum on plant growth and development and in communication with biotic factors (Folta and Maruhnich, 2007). Nevertheless, a few reports have shown effects of green light on plant–phyllosphere interactions and green light responsive genes must therefore be identified. Green light can reduce disease caused by *Pseudomonas cichorii* in tomato seedlings through the upregulation of defense-related genes (Nagendran and Lee, 2015). Green light can also be introduced in greenhouses in combination with traps to enhance capture of insects and pests (Chu et al., 2008; Ben-Yakir et al., 2012b; Jahan et al., 2014; Stukenberg et al., 2014; Park and Lee, 2017). This approach does not describe a direct impact of green light on plant-phyllosphere, but patterns of attraction to green light were described to be higher in whiteflies infected with *Tomato yellow leaf curl virus* than in non-infected individuals (Jahan et al., 2014). Green light-associated visual traps may therefore provide a solution to reduce virus transmission by vector insects.

### Colored Mulches and Nets

Shade netting, and colored cladding and mulches are options that have been considered in open and/or large fields and greenhouses. They may modify the spectral composition incident on a crop, beyond UV regions, with the potential to affect biotic interactions. Cladding may also create microclimates with altered air temperature, soil moisture, relative humidity, and total photosynthetically active radiation compared to open field conditions. Different materials are available and a proper selection may depend on factors such as cost, desired light spectra, the type of cultivated crop, or the climate of the region (Lamnatou and Chemisana, 2013a,b). Research has mostly focused on insects and parasites, and less on the phyllosphere. Reports have been mostly empirical and lack data at the molecular level. Selected colors may interfere with the capacity of a pest to locate its plant host, modify fly patterns, reduce attraction to plants, or deter landing on plant hosts (Ben-Yakir et al., 2012a). Effects on insects and pests can in turn affect leaf microbiomes. This relationship may be a guide for future approaches that aim at directly or indirectly targeting plant–phyllosphere interactions with colored netting and mulches. A cheap solution that may be of special interest to smallholder growers. It is, however, limited by the poor control of the light quality and intensity reaching the plants and the phyllosphere, and the fact that some microorganisms can growth both in light and dark conditions.

## Conclusion and Future Perspectives

The complexity of the phyllosphere and its interaction with plants is astonishing. This close association brings benefits for both parties and improves the growth and fitness of plants but it can also be deleterious for plants in the case of plant pathogens. These dynamic interactions are complex networks of living organisms that not only include plant and microorganisms but also go beyond to embrace invertebrates (i.e., parasites, insects, and others) as well as herbivorous animals. While microorganisms interacting with roots are influenced by the reduced availability of oxygen, higher osmotic pressure, and variable amounts of water and moisture, phyllosphere microorganisms have to face different challenges, as described in this review.

Light is a particular cue that determines survival and success. Most phyllosphere microorganisms are affected negatively to a lesser or greater extent by light, however, some groups use it as a complementary source of energy. The light spectrum is differentially sensed by microorganisms and by plant hosts. Light can be seen by microorganisms and by plants as discrete wavelengths in the human visible range up to far-red and including UV. Different narrow bandwidth wavelengths may have specific effects on both sides. The increasing knowledge of the regulatory systems supporting light responses is opening doors to simultaneously manipulate plant growth and microbial populations. Advancements in the field will strengthen strategies using selective lighting and precise light recipes in greenhouses and controlled environments to boost plant growth and production in conjunction with microbes.

There are gaps that remain to be filled (**Tables 1–4**). Most studies have focused on UV light, followed by blue, red, and far-red light. The increasing commercial availability of LEDs opens doors for additional, precisely controlled, tests targeting additional light colors and combinations. Narrow-bandwidth wavelengths that have been neglected include violet/high-energy blue, green, and yellow light. Light quality must not be the only concern. Light intensity and circadian rhythms also affect plant–phyllosphere interactions.

The complexity of plant–phyllosphere communications has often shown examples of specific interactions that depend on the plant host and the environmental conditions. It is common that laboratory models do not reflect behavior of plants in their natural habitats. Knowledge at the molecular level on model and non-model species is growing but further studies are needed.

The majority of reports have focused on detrimental organisms and less on beneficial organisms. Plant disease is certainly a concern for plant producers, but beneficial phyllosphere components contribute to plant fitness, including crop productivity. Future approaches must therefore address positive plant–phyllosphere interactions, according to individual plant hosts and environmental conditions. Studies have also focused on roles of defense-related plant hormones in light–phyllosphere interactions, but other hormones, namely auxin, must be assessed.

To consider the specificity of plant–phyllosphere interactions is of particular relevance in order to be able to address the diurnal or nocturnal habits of individual microorganisms. According to each situation it may beneficial to adjust photoperiods and/or consider the usage of night-break treatments.

Herbivores and their natural enemies are also responsive to light environments, which may indirectly impact phyllosphere communities. Some examples were given, particularly under UV light, but other regions of the light spectrum also affect herbivore behavior. Describing detailed examples of these responses went beyond the scope of this review. Nevertheless, it is evident that herbivore responses to light should be considered when establishing conditions for optimal plant–phyllosphere communications. These data remain highly empirical and need further research at the molecular level. Finally, a field that remains to be explored is the light-mediated communication between phyllosphere and rhizosphere.

## Author Contributions

JC and SC retrieved bibliographic information and wrote the manuscript.

## Conflict of Interest Statement

The authors declare that the research was conducted in the absence of any commercial or financial relationships that could be construed as a potential conflict of interest.
